# THE EMERGENCE OF THE DISORGANIZED/DISORIENTED (D) ATTACHMENT CLASSIFICATION, 1979–1982

**DOI:** 10.1037/a0038524

**Published:** 2015-02

**Authors:** Robbie Duschinsky

**Affiliations:** 1 Northumbria University

**Keywords:** attachment, developmental psychology, Ainsworth Strange Situation Procedure, anomaly, classification

## Abstract

This article examines the emergence of the concept of infant disorganized/disoriented attachment, drawing on published and archival texts and interviews. Since this new classification was put forward by Main and Solomon (1986), “disorganized/disoriented attachment” has become an important concept in clinical and social intervention contexts. Yet whereas Main and Solomon have often been misunderstood to have introduced disorganized/disoriented attachment in order to produce an exhaustive, categorical system of infant classifications, this article will suggest quite a different account. Attention will be paid to the emergence of disorganized attachment as a classification out of results and reflections in the late 1970s regarding the limits of an alarmed infant’s capacities for maintaining behavioral and attentional avoidance. In contrasting this interpretation of Main and Solomon’s work with current, widespread misunderstandings, the article will critically examine tendencies that have supported the reification and misapplication of the concept of disorganized/disoriented attachment.

Michael Rutter, a prominent commentator on attachment research, has described the discovery of the disorganized/disoriented attachment classification as one of the five great advances in psychology contributed by research in attachment. Yet Rutter has also raised a concern: The classification “undoubtedly identifies behavioral features of considerable theoretical and clinical significance, but the meaning of [the disorganized/disoriented attachment classification] remains rather unclear” (Rutter, Kreppner, & Sonuga-Barke, 2009, p. 532). Because statements may only yield their full meaning when placed in the intellectual context that was taken for granted by the researchers themselves when they were writing (Skinner, 2002), a historical investigation has the potential to specify the meaning of important psychological concepts, and in doing so, to unsettle contemporary assumptions. The argument of this article will be that, in interpretations of the addition of a “disorganized/disoriented attachment” classification by Main and Solomon (1986, 1990) to the Ainsworth & Wittig (1969) original tripartite classificatory system, Main’s earlier research and thinking about the potential relation between disorganization and both avoidance and ambivalence/resistance has fallen largely out of view. Without awareness of this earlier work, Main and Solomon have frequently been misunderstood as suggesting that their new category (a) represents heterogeneous chaos without logic or meaningful internal differentiation, and (b) completes a four-part and exhaustive typology of infant relationships, when added to the three Ainsworth infant attachment patterns.

The emergence of disorganized/disoriented attachment, and interpretations of Main and Solomon’s goals in proposing this new classification, are of interest as a case study within the rise of attention to child abuse in psychological research since the 1970s. It also has interest as a significant instance in which constructions of discrepant observational findings played a large role in theory change in developmental psychology. I am an outsider to the field of attachment research; my work has focused on the history and present-day role of psychological classifications within social policy, professional practice, and in psychiatric discourses. Elsewhere, I have engaged in a genealogy of disorganized/disoriented attachment as a discursive practice that stretches well beyond the attachment research community—for instance, analyzing its role within U.K. Early Intervention policy since 2010 (Duschinsky, Greco, & Solomon, 2015). My goal here, however, is to call into question the reification and misapplication of disorganized/disoriented attachment deployed in such policy and practice discourse, which invokes the authority of Main and Solomon.

The research presented here draws upon: the analysis of published texts, conference presentations, and doctoral theses; interviews with the researchers who led investigations of disorganized/disoriented attachment behavior, who have generously provided access to unpublished drafts, peer-review feedback, and correspondence; and the manuscripts and letters in the John Bowlby archive at the Wellcome Trust Archive in London. Attention to these documents and accounts from insiders in the attachment community are important for understanding the emergence of the disorganized/disoriented attachment classification, and also serve to highlight important differences between Main and Solomon’s reported goals in introducing the idea of “disorganized/disoriented attachment” and the way that, subsequently, this classification has generally been understood.

## Setting the Scene: Attachment Theory

The founder of attachment theory, the British psychoanalyst John Bowlby (1969), distinguished between the *attachment system* as a disposition that keeps an infant oriented toward closeness with their caregiver, and *attachment behavior* as the specific observable actions the infant uses to achieve proximity with the caregiver, particularly when distressed or alarmed. When activated, he anticipated that the attachment system would coordinate attachment behavior—in the form of signals and movements including crying, smiling, and crawling—to gain proximity, and thus protection and emotional support, from the caregiver. Influenced by ethology, Bowlby believed that the tendency for primate infants to develop attachments to familiar caregivers was the result of evolutionary pressures, as attachment behavior would facilitate the infant’s survival in the face of dangers such as predation, exposure to the elements, or attacks from conspecifics. Reflecting on the experiences of work with children who were evacuated during the war, Bowlby predicted that, because separation from their caregiver is a natural cue for danger for a human infant, under the injunction of the attachment system, experiences of separation would be met with protest and attempts to regain proximity.

Three patterns of attachment behavior were proposed by a Canadian colleague of Bowlby’s, Mary Ainsworth, then based at The John Hopkins University. Ainsworth and Wittig (1969) observed 26 infant–caregiver dyads in their Baltimore Strange Situation study. The Strange Situation Procedure was designed to use the cues of unfamiliarity and separation to elicit potential anxiety regarding the availability of the familiar caregiver. As such, the procedure aimed to mobilize the infant’s expectations about what happens when anxiety about the availability of the attachment figure has occurred in the past, and allowed a viewer to interpret these expectations from observed behavior.

In line with Bowlby’s (1969) predictions, Ainsworth & Wittig, (1969) (see Table 1) found that a majority of infants, classified as “Secure (B),” used the caregiver as a “safe base” from which to explore, protested at their departure, but sought the caregiver (attachment figure) upon his or her return. Here, a variety of attachment behavior—such as crying, smiling, and crawling—nonetheless seemed to coherently and directly express the demands of the attachment system for proximity and protection. In line with Bowlby’s prediction, Ainsworth’s home observations, as well as subsequent research, found that the caregivers for children classified as secure (B) were those most sensitive and responsive to the child’s attachment behavior. However, a minority of infants in Ainsworth’s middle-class sample showed little visible distress on separation or reunion with their caregiver. This appeared to contradict Bowlby’s theory. However, Ainsworth theorized that the apparently unruffled behavior of these infants was in fact a mask for distress—a hypothesis later evidenced through studies of the heart rate of avoidant infants (Sroufe & Waters, 1977). She termed this pattern of infant behavior “Avoidant (A),” because the infants avoided showing their distress to their attachment figure. Ainsworth concluded that when these infants had experienced alarm and distress in the past, they had learned that they should not communicate such feelings, as this would trigger rejection. A third pattern was termed “Ambivalent/Resistant (C),” and these infants often showed distress even before separation, and were frustrated and difficult to comfort on the caregiver’s return, seeming to distrust his or her availability even when the caregiver was present. Ainsworth’s former doctoral student, Mary Main (1979), theorized that the A and C patterns could be regarded as “conditional strategies” for optimizing, insofar as possible, the closeness with the caregiver impelled by the attachment system. In contrast to the direct proximity-seeking of the B infant in response to alarm, downplaying displays of attachment behavior in an avoidant (A) pattern could be regarded as an adaptation to a generally rebuffing caregiving environment; maximizing displays of distress and showing anger in an ambivalent/resistant (C) pattern could be adaptive in keeping the attention of a caregiver experienced as not reliable in responding to attachment signals.[Table-anchor tbl1]

In her doctoral research, conducted between 1968 and 1973, Main noticed the unclassifiable status of five Strange Situation narratives. As well as the measures required for her doctoral research, Main instructed her coders “to note each time that the toddler did anything which seemed odd to them”; this included “hand-flapping; echolalia; inappropriate affect; and other behaviors appearing out of context” (Main, 1977, pp. 70–71). She later recalls that “five out of 49 (10.2%) infants in her sample” were found to be “difficult to classify”: two of these infants were force-classified as secure, whereas three “were informally termed A-C infants within the laboratory” and classified either as A or C (Main & Solomon, 1990, p. 126). Main noted that two of these infants showed reunion behavior that combined an attempt to approach the caregiver with signs of fear and avoidance. One threw her hands in front of her face on reunion, whereas the other engaged in asymmetric hand slapping while creeping forward. Main “asked Mary Ainsworth as my dissertation advisor what to do. Characteristically cautious, but certain these infants were insecure, she recommended that for the time being (until more samples were collected and studied) we place them in Group A” (Mary Main, personal communication, August 10, 2012). Main (1973: 21) wryly noted in a footnote to her doctoral thesis that although this technique of anomalous cases was pragmatically useful, “Linnaeus might not approve.” The growing evidence over the years of unclassifiable infants raised “issues . . . critical to the use, validation, and interpretation of the Ainsworth system” (Main & Weston, 1981, p. 933).

In 1986, a new “disorganized/disoriented (D)” infant attachment classification was proposed for the Ainsworth Strange Situation Procedure by Mary Main and Judith Solomon, based at University of California, Berkeley. Often exhibited most strongly on reunion, but found in other episodes of the procedure as well, disorganized/disoriented behaviors suggest either a conflict between simultaneous dispositions to physically approach and to flee the caregiver—or seeming disorientation to the environment. Infant behaviors coded as disorganized/disoriented include overt displays of fear of the caregiver; contradictory behaviors or affects occurring simultaneously or sequentially; stereotypic, asymmetric, misdirected, or jerky movements; or freezing and apparent dissociation. In general, these behaviors occur only briefly, before the infant then enters back into one of the Ainsworth A, B or C attachment patterns. As such, all infants coded as disorganised/disoriented are also given a secondary A, B or C classification. The classification has been found to be a risk factor for later development (Sroufe et al., 1999). In the case of dissociative symptoms, for example, Carlson (1998) reported that a classification of disorganized/disoriented attachment in infancy had a .36 association with indices of dissociation in adolescence. A decade later, Dutra, Bureau, Holmes, Lyubchik, and Lyons-Ruth (2009) found that this association is unmediated by the experience of trauma.

After the initial presentation of protocols for coding D Strange Situation behavior in infants by Main and Solomon (1990), studies have examined caregiver behavior associated with behavior coded as disorganized/disorientated in the Strange Situation. In the same edited volume as Main and Solomon’s chapter, Main and Hesse (1990) proposed that frightening and frightened parental behavior could be the predominant mechanism producing disorganized/disoriented infant attachment. An association between frightening and frightened parental behaviour and the infant’s classification as D in the Strange Situation was supported by Schuengel, Bakermans-Kranenburg, and Van IJzendoorn (1999) as well as later studies. Extending and adding to this account, dissociative (Abrams, Rifkin, & Hesse, 2006) and helpless or withdrawing (Solomon & George, 1996; Lyons-Ruth et al., 2013) behaviors in a parent have also been found to predict an infant’s disorganized/disoriented attachment classification. As such, there are a plurality of factors that can increase the likelihood of infant disorganized/disoriented attachment, especially when they evoke feelings of fear that are not metabolized within the caregiving environment. Among these, a meta-analysis found that 48% of infants classified as D in the Strange Situation have been assessed by social services as experiencing abuse or neglect (van IJzendoorn, Schuengel, & Bakermans-Kranenburg, 1999). A parent’s ongoing experience of an anxiety disorder (Manassis, Bradley, Goldberg, Hood, & Swinson, 1994) or multiple forms of social and economic disadvantage (Cyr, Euser, Bakermans-Kranenburg, & Van IJzendoorn, 2010) have also been found to predict infant disorganized/disoriented attachment behavior. Furthermore, Solomon and George (2011) have documented that a chronic lack of regulation of the caregiving environment can predict disorganized/disoriented infant behavior in the Strange Situation Procedure. For example, major separation alone—in the absence of maltreatment (e.g., in care or divorce proceedings)—can increase the likelihood of a D classification of an infant in the Strange Situation Procedure.

Over the past decades, Kochanska and Kim (2013) have observed a “rapidly growing interest in disorganized attachment” from clinicians and policymakers, as well as researchers. Disorganized/disoriented attachment has become a central concern of research in developmental psychopathology, addressed in numerous articles and books. The concept has also seen wide use in a variety of clinical, intervention, and forensic contexts concerned with infant mental health; for example, assessments of disorganized/disoriented attachment from film recordings made of infant behavior have been used by social workers in investigating child maltreatment (Shemmings & Shemmings, 2014). Yet concerns have also been raised regarding the disorganized/disoriented attachment classification. Main and Solomon have been characterized as theorists of an exhaustive, categorical system bent upon “reducing complex human experience to typologies” (O’Shaughnessy & Dallos, 2009, p. 559). Likewise, Gaskins (2013) has alleged that Main and Solomon have offered the field an irredeemably flawed and dangerous concept, simply soaking up possible variation in human behavior beyond the Ainsworth patterns and treating it all as evidence of dysfunction. “The category is really just a residual one,” Gaskins argues, and rather than designating any meaningful phenomena, the existence of the classification “might be seen more productively as evidence of the inadequacy of the three attachment classifications” (Gaskins, 2013, p. 39). Such criticisms have some purchase on the way that the D classification has been used; however, to that degree, such use runs against the goals of those who proposed it. Main, Hesse, and Hesse (2011, p. 441) have criticized the “widespread” and “dangerous” presumption that infants can be divided into four categories of comparable status, and that any behavior besides the Ainsworth three patterns is disorganized and caused by frightening or abusive treatment by the parent. Solomon (personal communication, April 2, 2013) expresses particular concern that this misunderstanding is grounded in a mistaken narrative about what their intentions were in proposing the classification:

The reification of our work from its context—and a lack of awareness of the grounding of our ideas in the behavioral and theoretical contributions of Bowlby and Ainsworth—has lead readers to treat D as a category equivalent in kind to ABC, rather than recognizing it as a phenomenon that runs *orthogonal* to the basic Ainsworth patterns. (Solomon, personal communication, April 2013)

## Distinguishing Disorganization and Avoidance

At the heart of the emergence of the disorganized/disoriented attachment classification lies work at UC Berkeley in the late 1970s on the limits of the avoidant (A) attachment strategy. Main (1979, p. 640) emphasized that maintaining closeness with the caregiver in response to potential threats should be regarded as “the sine qua non for infant survival.” She argued that avoidant (A) attachment behavior in the Strange Situation Procedure should be regarded as “a conditional strategy, which paradoxically permits whatever proximity is possible under conditions of maternal rejection” by deemphasizing attachment needs (Main, 1979, p. 643). Yet Main was led to also consider the potential limits of young children to enact the emotion regulation necessary to maintain an avoidant conditional strategy. In 1979, Carol George completed her master’s thesis with Mary Main as her thesis advisor. The purpose of her study was to explore the correlates of attachment and physical child abuse, comparing the behaviors and interactions of nonabused children with children classified by social services as “abused” who were attending therapeutic day care in the San Francisco Bay Area. This was a period during which attention to child abuse by psychologists and the general public was seeing rapid growth (Hacking, 1991).

George (personal communication, September 13, 2012) recalls that “approach-avoidance behavior was prominent in this abuse sample,” with several children initially approaching their daycare caregiver, but then veering off with their eyes or face averted (see George & Main, 1979, p. 311). The prominence of approach avoidance in a maltreatment sample in day care raised the question of whether such behavior, when shown in the Strange Situation with parents, should be regarded as a coherent part of an avoidant attachment pattern or represented some disruption of this pattern:

Movements of avoidance in such situations do not merely “express” fear; by momentarily directing visual attention away from the attending partner they function to reduce the arousal of any disorganising, negative emotions/tendencies . . . [and ensure] the maintenance of a socially positive proximity. (George & Main, 1979, p. 315)

Whereas Main had previously assumed that maltreated children would show avoidance in the Strange Situation Procedure, in the late 1970s, she reconsidered this perspective as she reflected upon limits of infant’s capacity for maintaining a coherent avoidant strategy. She worked to theorize more precisely the mechanisms through which avoidance would defend an infant against distress and conflict. In a chapter largely composed during a research fellowship with Karin and Klaus Grossmann in Bielefeld in 1978, Main (1981) theorized that in avoidant infants, “this shift of attention is in fact only an attempt to reorganise or to maintain organization” (p. 683). Avoidant behavior is “a search for control when disorganisation threatens,” and is continuous with disorganization to the extent that it is ineffective at successfully diverting attention from the conflict between approach, withdrawal and anger (p. 685).

Like Block and Block, colleagues at Berkeley with whom she was in conversation, Main conceptualized infants as deploying strategies to respond to stressful situations and regulate their emotions; in the terms of Block and Block (1980, p. 48), “disorganization,” such as “immobilized, rigidly repetitive or behaviorally diffuse” flooding behaviors, could be expected when a child was experiencing “a difficulty in recouping” in the face of behavioral conflict and distress. In the Strange Situation, Main inferred that this “recouping” would mean some strategy for direct or conditional proximity-seeking. On the basis of this inference, Main (1981) was coming to theorize behavior characteristic of an “A” Strange Situation coding as only secondarily characterized by behavioral avoidance of the caregiver, and primarily as “avoidance of behavioral disorganisation” (p. 681) and the reduced ability to seek protection that a sustained state of emotional flooding would entail. Among the few to have noted this emphasis in Main’s account of avoidant attachment behavior, and writing before a reified account of her ideas crystallized, Bowlby (1980. p. 73) stated that Main conceptualized the infant displaying avoidant behavior is “avoiding any risk of being rebuffed and becoming distressed and disorganised; in addition he is avoiding any risk of eliciting hostile behavior from his mother.” The behavioral disorganization in the context of high distress and fear notable in abused children, “on a lesser scale have been observed in avoidant infants in normal samples”. According to Main, Bowlby (1984, p. 366) reports, the reason for this is that avoidance is an infant’s attempt to hold back the threat of losing control of their behavior to emotional flooding.

Main and Stadtman (1981) presented three studies in which behavior suggestive of a conflict of motivations (“conflict behavior”) was documented in infants classified as avoidant in the Strange Situation Procedure. Whereas in the stressful Strange Situation, “avoidance may function to modulate the painful and vacillating emotions,” Main and Stadtman (1981, p. 293) noted that “in less stressful situations we might expect to see the anger and conflict” that the infant had been too frightened to express in a stressful, unfamiliar environment. One of these studies was a reanalysis of Ainsworth’s records of home observations, which found that the infants who displayed avoidance on reunion in the Strange Situation Procedure, by contrast, visibly showed tension and conflict behaviors when rebuffed by their mother in the less stressful environment of the home. Main and Stadtman (1981, p. 301) noted, for example, that in one of the avoidant infants they observed that “in apparent direct response to the mother’s physical rejection, the infant grimaced, engaged in odd and empty laughter, kicked her feet many times in sudden peculiar tension movements, and engaged in stereotypies.” Such observations are inconsonant with any account drawing categorical distinctions between avoidant and disorganized/disoriented infants.

This article by Main and Stadtman (1981) has been almost entirely ignored since the 1980s. A likely reason for this neglect, proposed by Karin and Klaus Grossmann (personal communication, July 12, 2012), is that it is widely believed that Main disavowed any link between avoidance and conflict behavior when she announced the discovery of the disorganized/disoriented (D) classification (with Judith Solomon) and emphasized the role of frightened/frightening parental behavior (with Erik Hesse). The association between frightened/frightening parental behavior and disorganized/disoriented infant attachment had sufficient empirical support and a conceptually compelling quality, with the result that it magnetized the field’s curiosity. Thus, with the exception of Main herself (and a single chapter by Jacobvitz, Hazan, Zackagnino, Mesina, & Beverung, 2011), no subsequent researcher has interpreted Main and Stadtman (1981) as arguing for any more than the limited claim that the caregiver in avoidant dyads tends to rebuff their infant’s attachment behaviors. In fact, to the degree that other home-observation data replicate this finding, the article’s observations are disruptive of many widespread assumptions about disorganization, and have practical significance. A categorical distinction between avoidant and disorganized/disoriented infants has caused problems for later clinicians, research psychologists, and social welfare professionals, who are unaware that “avoidant babies often look like disorganised/disoriented babies in the home” (Main, personal communication, July 10, 2013). This is potentially an important issue for those who want to use disorganized/disoriented attachment behavior, for instance, within social services assessments (cf. Wilkins, 2012).

In fact, the three studies of Main and Stadtman provide evidence that, in the context of familiar situations in which stress is not high, direct expressions of behavioral conflict can be observed in precisely those infants classified as avoidant in the Strange Situation Procedure. The conclusions of Main and Stadtman might be placed together with Ainsworth’s finding that repeating the Strange Situation Procedure 2 weeks later caused all infants classified as avoidant to display conflict behaviors in accompaniment to proximity-seeking on reunion (Ainsworth, Blehar, Waters, & Wall, 1978, p. 221). Recalling this procedure, Mary Main (personal communication, March 5, 2013) reported that

the babies were simply too frightened not to approach their mother. Cancelling her study with her usual integrity, Mary Ainsworth said she was surprised that there was so little stability, but it was a coup for John Bowlby, since he expected approach under high stress or fear.

The dysregulation of avoidance can be expected when distress and fear overwhelms the infant’s capacity to regulate behavioral conflict. If subject to significantly more, or significantly less, distress than a standard Strange Situation Procedure, avoidant infants will directly show conflict behaviors when their attachment signals for contact are frustrated. If avoidance is to be regarded as “an organized yet incomplete shift in attention which is defensive in character, and which serves as an alternative to behavioral and emotional disorganisation” (Main & Stadtman, 1981, p. 293), this suggests that the avoidant strategy will only be activated when a lower threshold of stress is reached, but that it cannot be maintained beyond the breach of the floodgate represented by an upper threshold of distress and fear.

Considering the implications of these thresholds, Main and Stadtman wondered what could cause infants to show conflict behavior in the Strange Situation, when evolutionary theory appeared to suggest that greater apparent threat would elicit intensified attempts to achieve proximity or conditional proximity. They came to the conclusion that one sufficient, but not necessary, pathway to the expression of conflict behavior in the Strange Situation was caregiving that was itself in some way alarming—“A frightened child inevitably seeks the attachment figure as a haven of safety” (Main & Stadtman, 1981, p. 293)—but in cases when the attachment figure serves as a source of “alarm of any kind,” “at least two conflicting messages are received: to go away from, and to come toward, the haven of safety” (Main & Stadtman, 1981, p. 305). This pathway would produce, Main hypothesized, “an irresolvable and ultimately self-perpetuating conflict situation” between approach and withdrawal (Main & Stadtman, 1981, p. 293). Yet Main’s texts indicate that she did not presume that a conflict between attachment and alarm would be the only cause of displays of conflict behavior in the Strange Situation Procedure. Already by 1981, Main had theorized that a conflict between attachment and anger would also be capable of disrupting the smooth expression of the attachment system. When this conflict could be regulated by the infant, Main suggested that one possibility would be the angry subtype of ambivalent/resistant attachment behavior (C1). In this case, the attachment system would be punctuated, but not interrupted, by anger, as the resistant behaviors could be organized within the attachment system as a strategy to attract the attention of the infant’s caregiver. Main observed that the conflict between anger and attachment could flood out and interrupt the smooth expression of the attachment system, such that the infant would primarily be focused on expressing rage rather than oriented by their attachment system toward what proximity would be available from the caregiver. Ambivalent/resistant infants generally “could not be settled (from distress)” (Main & Weston, 1981, p. 934). This suggested to Main that the ambivalent/resistant pattern could sometimes become primarily an expression of anger or distress in the context of motivational conflict—and less an organized emotional and behavioral strategy to achieve the goal of the attachment system in attracting the attention of the caregiver. ^1^

By the early 1980s, Main appears to have assumed that the ambivalent/resistant (C) behaviors displayed by infants in the Strange Situation Procedure should be regarded as proximate to, and sliding into, a state of disorganized distress to the extent that an infant’s behaviors did not appear oriented in a functional way toward the caregiving environment. Offering the first published definition of the term *disorganized*, Main (1981, p. 683) stated that “behavior can be called disorganized when it vacillates between opposites without reference to changes in the environment, or when it appears repeatedly in an environment that does not call for it.” In this passage, Main gives two examples of disorganized behavior. The first is from the work of Robertson and Bowlby (1952): Their observational research on hospitalized children showed that “disorganized behavior appears in infants reunited with their mothers while still in the stages of protest or despair” (Main, 1981, p. 683). Bowlby’s discussion of disorganization as an intermission in behavioral and attentional regulation in the context of overwhelming emotion seems to have been an important anchor for Main’s later deployment of the term, and perhaps also supported its acceptance. For her second example of disorganized behavior, Main (1981, p. 683) refers to the behavior of “the ambivalent infants in the strange situation.” The definite article *the* here is suggestive that, for Main, disorganization could not simply be witnessed in some ambivalent/resistant (C) infants but, to varying degrees, in the C pattern in general.^2^ As such, disorganization was not conceptualized as categorically distinct from avoidance or ambivalence/resistance but as animating both—as the specter of losing control in the context of strategies to retain the availability of the caregiver and orientation to the environment.

## Disorganized/Disoriented Attachment Behavior

Having examined Main’s thinking by 1981 regarding the continuities between disorganization and the avoidant and ambivalent/resistant conditional strategies, the development of the quasi-interval scale of disorganized/disoriented behaviors can now be considered. This scale would prove significant for the formulation of D as an attachment classification—and its subsequent reification as an apparently exhaustive, residual addition to a taxonomy. In the mid-1970s, Main (1977, p. 70) foresaw “no way of fully defining in advance a set of potential ‘conflict’ behaviors.” Not all cases unclassifiable according to Ainsworth et al.’s (1978) coding protocols would necessarily be displaying conflict behavior, and—as we have seen—Main considered that conflict behavior would also partly characterize the A and C conditional strategies to the extent that these strategies were unsuccessful at modulating distress and conflict.

In 1977, Judith Solomon joined Mary Main’s lab following a graduate focus on ethology and comparative psychology. Main left soon after Solomon’s arrival for a 9-month visiting research fellowship in Bielefeld with Karin and Klaus Grossmann, and in her absence, instructed Solomon to learn how to classify Strange Situation procedures, guided by feedback that Main sent back to the laboratory (Judith Solomon, personal communication, September 6, 2012). The sample of recordings used by Solomon was Main’s Berkeley middle-class sample. Solomon began to compile detailed notes on cases she found difficult to classify, for discussion with Main on her return. At the same time, Solomon began to study a sample of maltreated infants in the Strange Situation with Carol George. This combination of dependence and independence on her advisor appears to have been significant in facilitating Solomon’s attention to discrepancies between the tapes she was viewing and Ainsworth’s coding protocols, as she was forced to make notes and think about these discrepancies with a delay before she could seek guidance on their meaning. Solomon noted a variety of behaviors discrepant with the Ainsworth coding protocols, which were particularly common in the maltreated sample: apparent signs of depression in infants; indications that an infant was attempting to muster an ABC strategy but failing to achieve this; infants initially approaching the caregiver but then veering off; and disoriented behaviors (e.g., the child leaves its arm hanging in the air). For example,

One little girl cried desperately for her father to return throughout the entire separation. At the moment of reunion she looked into his face and became completely silent, her chest heaving with the apparent effort of holding back her tears; in a moment she turned away to examine the toys at her feet; the remainder of the episode was followed by silent play, despite her father’s obvious attempts to interact. (Solomon, personal communication, September 6, 2012)

Though she was aware that there might well be no common factor linking together the diverse discrepant behaviors she was seeing, the fact that they were more common in the maltreated sample encouraged Solomon’s interest in inquiring further into their possible meanings in the Strange Situation. This further supported the existing emphasis on unclassifiable classes, proposed by Main and Weston (1981). Whereas during her year in Bielefeld, Main had agreed with Karin Grossmann to use “Not to Classify” for anomalous cases, Solomon recalls that by 1979, in her conversations with Main, she “was calling these cases ‘D,’ but without a clear sense of what that meant other than that these infants were insecure even when they showed some aspects of the secure patterns” (personal communication, September 6, 2012). The motivation of Main and Solomon to treat the discrepant behaviors as representing a new attachment classification appears to have been spurred by three factors. First, the addition of a new classification would mean that tapes showing disorganized or disoriented behaviors would no longer need to be force-classified as A, B, or C, increasing the construct and predictive validity of the Ainsworth classifications. Second, the addition of a new classification would draw attention to behaviors that seemed of particular interest, because they were more common in at-risk samples in a period of growing interest in child maltreatment. Third, the behaviors often had a jerky, contradictory, or disoriented quality, which disturbed the infant’s sequencing of movement or gesture in seeking their caregiver, and as such, despite their differences, suggested some disturbance of the expression of the attachment system in its capacity to smoothly coordinate behavior and attention.

As well as closely analyzing the unclassifiable tapes in her own sample, Main’s laboratory also began to collect unclassifiable tapes from other researchers working with high-risk samples, such as Mary J. O’Connor, Elizabeth Carlson, Leila Beckwith, and Susan Spieker. The result was that Solomon and Main could base their announcement of a new attachment classification on review of 100 low-risk and 100 high-risk dyads. In the winter of 1982, Main and Solomon begin work on their chapter announcing “discovery of a new, insecure-disorganised/disoriented attachment pattern.” Because of delays in the publication of the volume as a whole, the text waited until 1986 for publication. Indices for coding disorganized/disoriented attachment were published in 1990 (Main & Solomon, 1990).

## Description and Interpretation

As Solomon (personal communication, April 2, 2013) has recalled, the language of “category,” headlined in the 1986 announcement of “the discovery of a ‘D’ category of infant strange situation response” (Main & Solomon, 1986, p. 122) had the advantage of helping attract notice to an important phenomenon for researchers and clinicians—however, it also had the disadvantage of potentially reifing disorganization/disorientation. Following their introduction of “disorganized/disoriented attachment,” Main and Solomon have often been understood as assuming that their new classification (a) represents heterogeneous chaos without logic or meaningful internal differentiation, and (b) completes a four-part and exhaustive typology of infant relationships, when added to the three Ainsworth infant attachment patterns. To take an early example, Cummings was one of the editors of the volume within which Main and Solomon’s (1990) chapter was published. In his contribution to the volume, Cummings noted that “prediction of 6-year functioning was improved by treating D infants as a separate group” (Cummings, 1990, p. 317). However, he argued that “deviations from expected sequences do not constitute a sufficient criterion for classification” (p. 319). Against what he took to be Main and Solomon’s perspective, he proposed that D behaviors could not all be expected to reflect the same process of breakdown of “general functioning” (p. 316), and therefore that the category lacked coherence and meaning. A better criterion for “disorganization,” Cummings argued (p. 326), would be behaviors that do not appear to function to achieve *felt security*, which he argued should be regarded as the set goal of the infant’s attachment system rather than proximity with the caregiver. As such, the criticisms posed by Cummings illustrate the mistaken assumptions about Main and Solomon’s work that have subsequently been widespread in the literature.

These misunderstandings regarding Main and Solomon’s intentions in introducing the D classification cannot be maintained if attention is paid to the history of their 1-to-9 scale of disorganization/disorientation. In 1981,^3^ Main, working together with Berkeley graduate student Donna Weston, first formulated an unpublished Scale for Disordered/Disoriented Infant Behavior. This was not formulated in relation to the Ainsworth Strange Situation Procedure but as a measure for use in assessing infant behavior in a study of empathy (unpublished). In this procedure, the infant is encouraged to play with a friendly stranger dressed in a clown outfit, who then starts to cry when asked to leave the room. This scale was soon after used by Main and Stadtman (1981, p. 300) for picking out conflict behaviors in avoidant infants during a videotaped free-play session. A version of the scale was typed up by Main for Karin Grossmann in June 1982, during another visit to Germany. The manuscript shared with Grossmann is a 9-point scale, indexing behaviors including “stereotypies, episodes of immobilization, disoriented behavior, misdirected behavior, sudden disordered outbursts of activity, and sudden uninterpretable noises or movements” (unpublished manuscript, 1982). These are four of the seven indices of disorganization/disorientation (D) used by Main and Solomon (1990). It is missing “sequential” and “simultaneous” contradictory behavior patterns; “direct indices of apprehension regarding the parent” is also absent for the evident reason that the infant is reacting in this study to the clown, not their parent. As such, the thinking that went into the construction of this scale is in clear continuity with the Main and Solomon (1990) indices for coding disorganized/disoriented attachment in the Strange Situation (Table 2).[Table-anchor tbl2]

In the instructions for using this scale, Main (1982) specified that “behavior is not disordered simply because it is inappropriate, antisocial, asocial or because it appears infrequently”, noting, for instance, that anomalous behavior may occur “in contexts which make it readily understandable, [such] as, tongue-out and hand-flapping during excited ball-play, or lying prone and unmoving for a brief period when there are other indications of tiredness” (1982). Such specifications suggest that Main’s use of the term *disordered* was linked to the gap she faced between (visible) observations of behavior that showed no unitary and coherent strategy for seeking even conditional proximity on reunion in the Strange Situation Procedure, and the idea of (invisible) motivational conflict or disruption, which could be expressed behaviorally in any number of possible odd or out-of-context ways. Attempting to discern which among anomalous behaviors could be best regarded as expressions of conflict or disruption, in the 1982 manuscript behavior is identified by Main as *disordered* based on the “extent to which such behavior may be indicative of difficulties in functioning” of the attachment system, for instance, by virtue of lacking either “orientation” or “purpose.” In the discussions between Main and Solomon, however, the term *disorganized* was used rather than *disordered* (though the latter term makes a cameo return in Footnote 6 of Main & Solomon, 1990). Main’s husband and collaborator, Erik Hesse (personal communication, January 8, 2013), reported that the consideration was that “‘disordered’ sounded pejorative.”

Because it was not a residual category used to soak up any and all heterogeneity, Main and Solomon therefore did not equally weight the behaviors that index disorganization/disorientation or expect that they would have the same meaning. The 1-to-9 scale was introduced as a device for assessing the degree and type of disorganized/disoriented behavior as a measure of *interpretive certainty* regarding conflict or disruption among behavioral tendencies in the Strange Situation Procedure—understood, in turn, to reflect a parallel disruption of the infant’s representations of the caregiver that integrate the attachment system (see Main, Kaplan, & Cassidy, 1985, p. 75). It would only be at the highest ends of the scale that this certainty could be translated into an assessment of degree of disorganization/disorientation, because of the pervasiveness or intensity of the behavior. This can be illustrated with the case of infant hand-to-mouth reunion behaviors in the Strange Situation. The hand-to-mouth gesture on reunion is an oddly privileged “direct index of disorganised attachment” for Main and Solomon (1990, p. 139; illustrated on p. 145): It is the only distinct behavior that is framed as directly *instantiating*, rather than merely pointing to, the construct of *disorganization*. On its own, with no other behavioral signs, an infant’s hand-to-mouth behavior on reunion is situated as “usually sufficient for D category placement” (p. 140). Solomon (personal communication, September 2012) relates that she and Main “pondered together the meaning of these ‘hand-to-mouth’ gestures. I used to walk around imagining the contexts in which I and most others use the gesture, which is often at moments of indecision or conflict. For me what was more striking was the way in which toddlers were attempting to swallow or smother distress by covering their mouths.”

Because D is coded when a viewer infers from visible behavior a disruption of the (invisible, posited) attachment system, this sharply raises the general problem of inferential reasoning in relation to observable behavior. Main and Solomon understood that hand-to-mouth reunion behavior could have a variety of functions and would not necessarily represent a breakdown of strategic functioning. However, the behavior was placed as an index of disorganization when it occurs on reunion with the caregiver, because they believed that, in such circumstances, it likely either directly (via fear) or indirectly (via confusion or constriction) suggests the presence of conflict in or dysregulation of the operation of the attachment system^4^—though the viewer can never know for sure.

Though Hesse and Main (2000) would go on to describe disorganization/disorientation as a “collapse in behavioral and attentional strategies,” this is a statement about the (invisible) attachment behavioral system and should not be regarded necessarily as a description of observable attachment behavior. For example, overt and behaviorally coherent displays of fear, such as hiding from the returning parent under a chair, are coded as *disorganized/disoriented* because they indicate a dysregulation of the attachment system that would otherwise be expected to gear a scared child to achieve proximity to the caregiver. Unfortunately, the Hesse and Main reference to a “collapse” of strategy has been widely misunderstood, with many presuming that disorganization/disorientation as a “collapse in behavioral and attentional strategies” always means a pervasive and chaotic breakdown of observable behavior (see, e.g., Parke & Clarke-Stewart’s, 2011 textbook)—rather than a disruption to the behavioral and attentional components of the imputed attachment system as it works to achieve its set goal. According to the coding instructions in Main and Solomon (1990), only infants with a score of 8 or 9 out of 9 might be expected to show so pervasive a disruption of attachment that the result is behavioral or attentional chaos in the Strange Situational Procedure. In subsequent research, infants with such high scores are rare. Infants with a score of 5 to 7, which is sufficient for a D classification, rather show some disruption of the behavioral or attentional components of the attachment system, which is considered sufficient for a D classification on the basis that the specific behavior is repeated, intense, extended in duration, or occurs right at the moment of reunion. The rest of the behavior shown by these infants in the Strange Situation may otherwise be coherently sequenced and oriented in relation to their caregiver and the environment.

Against the idea of disorganized/disoriented behavior as mere chaotic dysfunction, Main specifically states that this behavior may be a logical, adaptive response to the infant’s caregiving environment. Influenced by ethological observations that expressions of behavioral conflict may nonetheless be adapted (at an individual level) to the environment (e.g., Hinde, 1966, p. 276), Main (1990, p. 56) explained that “the individual’s behavior in a given situation may be an indication of what is most adaptive in that situation, but this does not inform us as to whether or not the individual is experiencing any secondary, counterwilled tendency.”

Consider two infants (cases from Main’s doctoral sample) who showed behaviors later classified as D: One flung her hands in front of her face on seeing her caregiver, whereas the other engaged in asymmetrical floor-slapping. The behavior of the first infant might well be regarded as adaptive to a threatening caregiving context, whereas the behavior of the second infant does not readily appear adaptive at an individual level.

## Conclusion

There is a wide tendency across psychological discourses to mummify classifications, especially when they are regarded as having predictive validity (Brown & Stenner, 2009). In the case of disorganized/disoriented attachment, this process appears to have been supported by two further factors. First, Main and Solomon’s (1986) own narrative and formulation of the new classification scaffolded some misunderstanding. For instance, Main and Solomon (1986) initially headlined a “new category” of attachment behavior, and this announcement was not read in the context of Main’s other work that linked the process of disorganization to avoidance and ambivalence/resistance. Second, the rise of “child abuse” as a recognized social problem during the period created pressures in clinical and welfare settings to find a tool and concept for distinguishing between maltreating and adequate parenting. It is my hope that, in attending to the goals of Main and Solomon in introducing the disorganized/disoriented attachment classification, this can help counter and qualify essentialist deployments of the concept, documented, for instance, in Duschinsky et al. (2015), which invoke Main and Solomon names as authority and justification. I would also be pleased if this critical historical analysis could help counter tendencies within the attachment research community to reify “disorganization/disorientation,” which have been observed to have “moved researchers away from attempting to examine patterns in the attachment behavior of disorganized infants” (Padrón, Carlson & Sroufe, 2014, p. 202). Close attention to the context of Main and Solomon’s introduction of “disorganized/disoriented attachment” indicates that the D classification was not intended to capture all anomalous behavior as indicating a unitary dysfunction in the mental health of the infant, but to scale the degree of certainty in the coder that the (visible) behavior under observation represented a disruption of an infant’s (invisible, imputed) attachment system.

Rather than as the innovator of an exhaustive and residual category for exceptions to the Ainsworth et al. (1978) protocols, attention to the work of Main in the late 1970s and early 1980s suggests that in introducing the concept of “disorganized/disoriented attachment,” Main and Solomon should rather be regarded as theorists of the implications at the level of behavior of *expressions* and *circumventions* of dysregulation of the attachment system. The diversity of possible expressions and circumventions of disorganization meant specifically that the

discovery of the D category of infant Strange Situation behavior rested on an unwillingness to adopt the “essentialist” or “realist” position regarding the classification of human relationships. It was based on the presumption that both individuals and relationships are unique and that they have a higher “reality” than any classification can fully encompass. (Main et al., 1985, p. 99)

Considered in this light, the goal of Main and Solomon can be regarded as an attempt to raise attention to the potential significance of visible behaviors that appeared to suggest some degree of disruption of the imputed, (invisible) attachment system. At the heart of their perspective lies the idea that “there exist species-wide abilities that are not part of the attachment system itself, but can, *within limits*, manipulate (either inhibit or increase) attachment behavior in response to differing environments” (Main et al., 2005, p. 256); indeed, Solomon’s present work at the Universität Wien is on the species specificity of such abilities. As such, their work activates a possibility, noted by Kierkegaard (1843/2009, p. 78), that “when one really wants to study the universal, one need only examine a legitimate exception, because it will present everything.” Rather than as essentialist innovators of an exhaustive and residual category for exceptions to the Ainsworth protocols, in their work on disorganized/disoriented infant attachment, Main and Solomon should be regarded primarily as theorists of expressions and circumventions of dysregulation of the attachment system.

## Figures and Tables

**Table 1 tbl1:** The Ainsworth Strange Situation Classifications

Attachment classification	Strange-situation behavior
**A**	Lower proximity-seeking and contact-maintaining on reunion than B or C, together with some proximity-avoiding behaviors. The infant’s behavior, attention, and affect are integrated in a coherent way to downplay the communication of distress and keep focus away from the caregiver (e.g. by attention to the toys).
A1	Lowest proximity-seeking and contact-maintaining on reunion than B or C; strongest proximity-avoiding behaviors.
A2	Low to moderate proximity-seeking on reunion. Marked proximity-avoiding behaviors.
**B**	Strong proximity-seeking and contact-maintaining on reunion compared with A. Low contact-resisting compared with C. The infant’s behavior, attention, and affect integrate in a coherent way, which allows distress to be communicated to the caregiver and assuaged, allowing the child to then return calmly to play.
B1	Weak proximity-seeking and contact-maintaining. Weaker proximity-avoiding behaviors than A1. Strong communication and affective sharing with their caregiver from a distance. Conceptualized as intermediate between A and B infants.
B2	Low to moderate proximity-seeking and marked proximity-avoiding on first reunion. But then strong proximity-seeking and contact-maintaining on second reunion.
B3	Strong proximity-seeking and contact-maintaining on reunion. No contact-resisting or proximity-avoiding.
B4	Some proximity-seeking and contact-maintaining prior to separation from the caregiver. Strong proximity-seeking and contact-maintaining on reunion. Some contact-resisting.
**C**	Marked contact-resisting behavior. The infant’s behavior, attention, and affect integrate in a coherent way, which strongly communicates their distress and frustration to the caregiver.
C1	Strong proximity-seeking and contact-maintaining on reunion. Strong contact-resisting behavior punctuates the contact maintaining, as the child switches between communicating distress and a desire for contact, anger, and a desire to be put down.
C2	Weak proximity-seeking but moderate to strong contact-maintaining, particularly on second reunion. Moderate contact-resisting.
*Note.* Ainsworth’s (1984) interactive behavioral measures, elaborated in detail in *Patterns of Attachment*:	
(a) proximity-seeking = the intensity, duration, and degree of success of the infant’s attempts to make contact with their caregiver, particularly where this occurs at reunion;	
(b) contact-maintaining = the intensity, duration, and degree of success of the infant’s attempts to keep contact with their caregiver once it has been achieved;	
(c) proximity-avoiding = the intensity and duration of behaviors that direct attention away from the caregiver as he or she approaches on reunion, such as averting the face;	
(d) contact-resisting = the intensity and duration of behaviors that signal anger and a desire to be put down from contact with the caregiver, such as pushing away.	

**Table 2 tbl2:** Indices of Disorganization/Disorientation

I. Sequential display of contradictory behavior patterns
II. Simultaneous display of contradictory behavior patterns
III. Undirected, misdirected, incomplete, and interrupted movements
IV. Stereotypies, asymmetrical movements, mistimed movements, and anomalous postures
V. Freezing, stilling, and slowed movements and expressions
VI. Direct indices of apprehension regarding the parent
VII. Direct indices of disorganization or disorientation
*Note.* From Main & Solomon (1990).

## Footnotes

1 Indeed, Ainsworth (1984, p. 581) would later observe, reflecting on ambivalent/resistant behavior in her Baltimore sample, that “to mingle intense anxiety and intense anger toward an attachment figure occasions severe conflict . . . when it is directed toward attachment figures, it would seem to be dysfunctional because of the risk of alienating them, but it can be functional and probably began thus.”

2 In line with this conclusion, Main and Hesse (1990, p. 179) later stated that “the ambivalent pattern seems much the least well organised. This may be in part because a majority of C infants are in fact disorganised.”

3 Though Main et al. (2011) state that the construction of the first scale for assessing disorganized behavior occurred in 1979, Mary Main and Erik Hesse have corrected this to 1981 (personal communication, September 13, 2012).

4 As a point of comparison, one might, for example, think of Agnes, in Kurt Lewin’s (1930) famous early film observations, who displayed hand-to-mouth behavior upon trampling her younger brother in her haste to escape a bully.

## References

[c1] Abrams K. Y., Rifkin A., & Hesse E. (2006). Examining the role of parental frightened/frightening subtypes in predicting disorganized attachment within a brief observational procedure. Development and Psychopathology, 18, 345–361 10.1017/S0954579406060184 16600058

[c2] Ainsworth M. D. (1984). Attachment In Endler N. S. & McVicker Hunt J. (Eds.), Personality and the behavioural disorders (2nd ed., pp. 559–602). New York, NY: Wiley.

[c3] Ainsworth M. D., Blehar M., Waters E., & Wall S. (1978). Patterns of attachment: A psychological study of the Strange Situation. Hillsdale, NJ: Erlbaum.

[c4] Ainsworth M., & Wittig B. (1969). Attachment and exploratory behavior of one-year-olds in a strange situation In Foss B. (Ed.), Determinants of infant behavior, IV (pp. 113–136). London, UK: Methuen.

[c6] Block J., & Block J. (1980). The role of ego-control and ego-resiliency in the organisation of behaviour In Collins W. A. (Ed.), Minnesota Symposia on Child Psychology: Vol. 13 Development of cognition, affect, and social relations (pp. 39–101). Hillsdale, NJ: Erlbaum.

[c7] Bowlby J. (1969). Attachment (1st ed.). London, UK: Penguin.

[c8] Bowlby J. (1980). Loss. London, UK: Penguin.

[c9] Bowlby J. (1984). Attachment (2nd ed.). London, UK: Penguin.

[c10] Brown S. D., & Stenner P. (2009). Psychology without foundations. London, UK: Sage.

[c11] Carlson E. A. (1998). A prospective longitudinal study of attachment disorganization/disorientation. Child Development, 69, 1107–1128 10.1111/j.1467-8624.1998.tb06163.x 9768489

[c12] Cummings E. M. (1990). Classification of attachment on a continuum of felt-security In Greenberg M. T., Cicchetti D., & Cummings E. M. (Eds.), Attachment in the preschool years (pp. 311–338). Chicago, IL: University of Chicago Press.

[c13] Cyr C., Euser E. M., Bakermans-Kranenburg M. J., & Van IJzendoorn M. H. (2010). Attachment security and disorganization in maltreating and high-risk families: A series of meta-analyses. Development and Psychopathology, 22, 87–108 10.1017/S0954579409990289 20102649

[c14] Duschinsky R., Greco M., & Solomon J. (2015). Wait up! Attachment and sovereign power. International Journal of Politics Culture and Society. Advance online publication 10.1007/s10767-014-9192-9 PMC5593123 28904425

[c15] Dutra L., Bureau J. F., Holmes B., Lyubchik A., & Lyons-Ruth K. (2009). Quality of early care and childhood trauma: A prospective study of developmental pathways to dissociation. Journal of Nervous and Mental Disease, 197, 383–390 10.1097/NMD.0b013e3181a653b7 19525736 PMC2697443

[c16] Gaskins S. (2013). The puzzle of attachment In Quinn N. & Mageo J. M. (Eds.), Attachment reconsidered: Cultural perspectives on a Western theory (pp. 33–66). London, UK: Palgrave.

[c17] George C., & Main M. (1979). Social interactions of young abused children: Approach, avoidance, and aggression. Child Development, 50, 306–318 10.2307/1129405 487875

[c18] Hacking I. (1991). The making and molding of child abuse. Critical Inquiry, 17, 253–288 10.1086/448583

[c19] Hesse E., & Main M. (2000). Disorganized infant, child, and adult attachment: Collapse in behavioral and attentional strategies. Journal of the American Psychoanalytic Association, 48, 1097–1127 10.1177/00030651000480041101 11212184

[c20] Hinde R. (1966). Animal behavior. New York, NY: McGraw-Hill

[c21] Jacobvitz D., Hazan N., Zackagnino M., Mesina S., & Beverung L. (2011). Frightening maternal behaviour, infant disorganisation, and risks for psychopathology In Cicchetti D. & Roismann G. (Eds.), The origins and organization of adaptation and maladaptation (pp. 283–322). New York, NY: Wiley.

[c22] Kierkegaard S. (2009). Repetition and philosophical crumbs (Mooney E., Trans.). Oxford, UK: Oxford University Press (Original work published 1843)

[c23] Kochanska G., & Kim S. (2013). Early attachment organization with both parents and future behavior problems: From infancy to middle childhood. Child Development, 84, 283–296 10.1111/j.1467-8624.2012.01852.x 23005703 PMC3530645

[c24] Lewin K. (1930). Expression movements in children In Stern W. (Ed.), Psychology of early childhood (6th ed., pp. 573–582). London, UK: Allen & Unwin.

[c25] Lyons-Ruth K., Bureau J. F., Easterbrooks M. A., Obsuth I., Hennighausen K., & Vulliez-Coady L. (2013). Parsing the construct of maternal insensitivity: Distinct longitudinal pathways associated with early maternal withdrawal. Attachment & Human Development, 15, 562–582 10.1080/14616734.2013.841051 24299135 PMC3861901

[c26] Main M. (1973). Exploration, play, and cognitive functioning (Unpublished doctoral dissertation). The John Hopkins University, Baltimore, MD.

[c27] Main M. (1977). Analysis of a peculiar form of reunion behaviour seen in some day-care children In Webb R. (Ed.), Social development in childhood (pp. 33–78). Baltimore, MD: John Hopkins University Press.

[c28] Main M. (1979). The ultimate causation of some infant attachment phenomena: Further answers, further phenomena, and further questions. Behavioral and Brain Sciences, 2, 640–643 10.1017/S0140525X00064992

[c29] Main M. (1981). Avoidance in the service of attachment In Immelman K., Barlow G., Petrinovitch L., & Main M. (Eds.), Behavioral development (pp. 651–693). Cambridge, UK: Cambridge University Press.

[c54] Main M. (1982). Scale for disordered, disoriented and undirected behaviors—Developed for clown session, unpublished manuscript.

[c30] Main M. (1990). Cross-cultural studies of attachment organization: Recent studies, changing methodologies, and the concept of conditional strategies. Human Development, 33, 48–61 10.1159/000276502

[c33] Main M., & Hesse E. (1990). Parents’ unresolved traumatic experiences are related to infant disorganized attachment status In Greenberg M. T., Cicchetti D., & Cummings E. M. (Eds.), Attachment in the preschool years (pp. 161–181). Chicago, IL: University of Chicago Press.

[c34] Main M., Hesse E., & Hesse S. (2011). Attachment theory and research: Overview with suggested applications to child custody. Family Court Review, 49, 426–463 10.1111/j.1744-1617.2011.01383.x

[c55] Main M., Hesse E., & Kaplan N. (2005). Predictability of attachment behavior and representational processes at 1, 6, and 19 years of age: The Berkeley Longitudinal Study In Grossmann K. E., Grossmann K., & Waters E. (Eds.), Attachment from infancy to adulthood: the major longitudinal studies (pp. 245–304). New York: Guilford Press.

[c35] Main M., Kaplan N., & Cassidy J. (1985). Security in infancy, childhood, and adulthood: A move to the level of representation. Monographs of the Society for Research in Child Development, 50, 66–106.

[c36] Main M., & Solomon J. (1986). Discovery of a new, insecure-disorganized/disoriented attachment pattern In Yogman M. & Brazelton T. B. (Eds.), Affective development in infancy (pp. 95–124). Norwood, NJ: Ablex.

[c37] Main M., & Solomon J. (1990). Procedures for identifying infants as disorganised/disoriented during the Ainsworth Strange Situation In Greenberg M. T., Cicchetti D., & Cummings E. M. (Eds.), Attachment in the preschool years (pp. 121–160). Chicago, IL: University of Chicago Press.

[c38] Main M., & Stadtman J. (1981). Infant response to rejection of physical contact by the mother: Aggression, avoidance, and conflict. Journal of the American Academy of Child Psychiatry, 20, 292–307 10.1016/S0002-7138(09)60990-0 7264108

[c39] Main M., & Weston D. (1981). The quality of the toddler’s relationship to mother and to father: Related to conflict behavior and the readiness to establish new relationships. Child Development, 52, 932–940 10.2307/1129097

[c40] Manassis K., Bradley S., Goldberg S., Hood J., & Swinson R. P. (1994). Attachment in mothers with anxiety disorders and their children. Journal of the American Academy of Child & Adolescent Psychiatry, 33, 1106–1113 10.1097/00004583-199410000-00006 7982861

[c56] O’Shaughnessy R., & Dallos R. (2009). Attachment research and eating disorders: A review of the literature. Clinical Child Psychology & Psychiatry, 14, 559–574. 19759074 10.1177/1359104509339082

[c41] Padrón E., Carlson E. A., & Sroufe L. A. (2014). Frightened versus not frightened disorganized infant attachment: Newborn characteristics and maternal caregiving. American Journal of Orthopsychiatry, 84, 201–208 10.1037/h0099390 24826936 PMC4085543

[c42] Parke R. D., & Clarke-Stewart A. (2011). Social development. New York, NY: Wiley.

[c43] Robertson J., & Bowlby J. (1952). Responses of young children to separation from their mothers. Courier of the International Children’s Centre, Paris, 2, 131–140.

[c44] Rutter M., Kreppner J., & Sonuga-Barke E. (2009). Emanuel Miller Lecture: Attachment insecurity, disinhibited attachment, and attachment disorders: Where do research findings leave the concepts? Journal of Child Psychology and Psychiatry, 50, 529–543 10.1111/j.1469-7610.2009.02042.x 19298474

[c45] Schuengel C., Bakermans-Kranenburg M. J., & Van IJzendoorn M. H. (1999). Frightening maternal behavior linking unresolved loss and disorganized infant attachment. Journal of Consulting and Clinical Psychology, 67, 54–63 10.1037/0022-006X.67.1.54 10028209

[c46] Shemmings D., & Shemmings Y. (2014). Assessing disorganized attachment behaviour in children. London, UK: Jessica Kingsley.

[c47] Skinner Q. (2002). Visions of politics. Cambridge, UK: Cambridge University Press.

[c48] Solomon J., & George C. (1996). Defining the caregiving systems: Toward a theory of caregiving. Infant Mental Health Journal, 17, 183–197 10.1002/(SICI)1097-0355(199623)17:3<183::AID-IMHJ1>3.0.CO;2-Q

[c58] Solomon J., & George C. (2011). The Disorganised Attachment-Caregiving System In Solomon Judith & George Carol (Eds.), Disorganized attachment & caregiving (pp. 25–51), NY: Guilford Press.

[c50] Sroufe A., & Waters E. (1977). Attachment as an organizational construct. Child Development, 48, 1184–1199 10.2307/1128475

[c59] Sroufe L. A., Carlson E. A., Levy A. K., & Egeland B. (1999). Implications of attachment theory for developmental psychopathology. Development and Psychopathology, 11, 1–13. 10208353 10.1017/s0954579499001923

[c51] van IJzendoorn M. H., Schuengel C., & Bakermans-Kranenburg M. J. (1999). Disorganized attachment in early childhood: Meta-analysis of precursors, concomitants, and sequelae. Development and Psychopathology, 11, 225–249 10.1017/S0954579499002035 16506532

[c52] Wilkins D. (2012). Disorganised attachment indicates child maltreatment: How is this link useful for child protection social workers? Journal of Social Work Practice, 26, 15–30 10.1080/02650533.2011.598228

